# Prevalence and socioeconomic determinants of food insecurity among Venezuelan migrant and refugee urban households in Peru

**DOI:** 10.3389/fnut.2023.1187221

**Published:** 2023-06-15

**Authors:** Akram Hernández-Vásquez, Rodrigo Vargas-Fernández, Fabriccio J. Visconti-Lopez, Juan Pablo Aparco

**Affiliations:** ^1^Centro de Excelencia en Investigaciones Económicas y Sociales en Salud, Vicerrectorado de Investigación, Universidad San Ignacio de Loyola, Lima, Peru; ^2^Facultad de Ciencias de la Salud, Universidad Científica del Sur, Lima, Peru; ^3^Sociedad Científica de Estudiantes de Medicina – UPC, Facultad de Ciencias de la Salud, Lima, Peru; ^4^Centro Nacional de Alimentación y Nutrición, Instituto Nacional de Salud, Lima, Peru

**Keywords:** food insecurity, refugees, emigrants and immigrants, prevalence, cross-sectional studies, Peru, Venezuela

## Abstract

**Introduction:**

Food insecurity (FI) is a public health problem affecting many regions of the world. In Venezuela, the political, social and economic situation experienced since 2010 has caused a mass migration of its population to other countries, including Peru, which, in turn, may have limited access to and availability of food leading to a high nutritional burden in this population. The objective of this study was to determine the prevalence and analyze the determinants of FI in the households of Venezuelan immigrants in Peru.

**Methods:**

A cross-sectional study was conducted using the “Encuesta Dirigida a la Población Venezolana que Reside en el País” (ENPOVE 2022). The dependent variable was moderate–severe FI (yes/no), which was constructed from an eight-item Food Insecurity Experience Scale (FIES) to measure FI at the household level. Poisson log generalized linear regression models were fitted to assess the association between the independent variables and FI. In addition, the reliability of the FIES as a tool for measuring food insecurity in the target population was determined.

**Results:**

A total of 3,491 households with Venezuelan migrants and refugees were included in the analysis. We found that 39.0% of Venezuelan immigrant households in Peru experienced moderate–severe FI. The determinants of FI included socio-demographic characteristics of the household head, and economic and geographical characteristics of the household. Regarding the FIES, we found that the inclusion of 7 of the 8 items had adequate internal consistency and its items assessed the same latent range.

**Discussion:**

This study highlights the need to identify determinants associated with FI to design strategies that mitigate the consequences of health crises and strengthen regional food systems, making them more sustainable. Although several studies have evaluated the prevalence of FI in Venezuelan migrant populations in other countries, this study is the first to evaluate the determinants of FI in Venezuelan immigrant households in Peru.

## 1. Introduction

Food insecurity (FI) is defined as the situation in which a person lacks constant availability and access to sufficient food to lead an active and healthy life (1). FI is a public health problem in various regions of the world, especially among people living in poverty and vulnerability (1). Although FI is included in the Sustainable Development Goals (SDGs), specifically in SDG 2 to eradicate hunger and all forms of malnutrition by 2030, it is estimated that more than 900 million people worldwide experienced FI in 2021 (2).

It is estimated that around 2010, Venezuela’s migratory profile started changing from that of a destination country to a country of origin. However, it is only since 2014 that Venezuelan emigration started reaching dramatic levels. In fact, the highest migration figures of the Venezuelan population were observed from 2016 around the world, with an exponential increase in the last five years (3–5). More than 7 million refugees and migrants from Venezuela have left their country in search of safety and stability. The vast majority, almost six million people, live in 17 countries in Latin America and the Caribbean. One of the most common destinations for Venezuelan migrants is Peru (only second to Colombia), where more than one million people have migrated in the last 10 years (6, 7). However, despite having left their home country due to precarious living conditions (e.g., food shortages, illegal food trade, unauthorized distribution networks, and high food prices), many of these migrants continue to suffer the consequences of FI due to the difficulties they face in accessing adequate and nutritious food in their destination country (8, 9).

The causes of FI in immigrant households are multiple and complex, ranging from lack of economic resources to buy food to lack of access to basic health and education services (10, 11). One report described some relevant indicators that explain the FI of Venezuelan migrants in Peru, including the lack of formal employment and precarious work, which limit their income and therefore their ability to purchase food. Additionally, the number of individuals residing in each household is a factor that also affects FI (12). However, institutions such as the United Nations High Commissioner for Refugees and the International Organization for Migration are carrying out various tasks (e.g., humanitarian assistance, protection, socioeconomic and cultural inclusion, integration, and reduction of xenophobia, among others) to improve the living conditions of Venezuelans in Peru (13).

On the other hand, the different dietary habits of the migrant population, the lack of nutritional education and information about healthy eating habits, as well as the availability of food at unaffordable prices, exacerbate FI in this migrant population, especially after the COVID-19 pandemic that worsened this situation (14). In addition, discrimination and xenophobia also play an important role in the availability, access, and consumption of food for adequate nutrition, as many Venezuelan migrants are excluded from jobs, housing, and basic public services due to their migratory status (10). Identifying modifiable factors associated with FI can serve as a basis for designing strategies that mitigate the consequences of disruptions caused by health crises (e.g., the COVID-19 pandemic) and the resulting FI and strengthen regional food systems, making them more sustainable (15).

Although several studies have evaluated the prevalence of FI in Venezuelan migrant populations in other countries, to our knowledge no study has evaluated the determinants of this problem in Peru (9, 10, 16). Therefore, the objective of this study was to determine the prevalence and analyze the determinants of FI in the households of Venezuelan immigrants in Peruvian territory, according to the “Encuesta Dirigida a la Población Venezolana que Reside en el País” (ENPOVE 2022).

## 2. Materials and methods

### 2.1. Data source and sampling

The analysis for this study was conducted based on data extracted from the ENPOVE 2022. The survey was carried out by the National Institute of Statistics and Informatics (INEI - acronym in Spanish) of Peru and collected representative data on household and individual information on the Venezuelan refugee and migrant population in Peru (17, 18). ENPOVE 2022 was conducted between February and March 2022, and it is the second version of the survey first conducted in 2018 (17).

The ENPOVE 2022 uses a probabilistic, stratified and independent sampling technique in urban areas of the main cities of Peru [Tumbes, Piura, Chiclayo, Trujillo, Chimbote, Ica, Arequipa, Metropolitan Lima (which includes the Constitutional Province of Callao)] (17). A sample frame of 236,074 households of the Venezuelan population in Peru was constructed and a total of 195,710 households were obtained (82.9% of the total number of households with Venezuelan population at the national level). The total sample size was 3,680 households with Venezuelan population with 12,487 participants usually residing in private and collective households. Face-to-face interviews were carried out during the months of survey (17, 18). Further specifications on the sample design, procedures and data collection can be found in the ENPOVE 2022 technical report (17).

### 2.2. Variables and measurements

#### 2.2.1. Household food insecurity measurement

The ENPOVE 2022 used the Food Insecurity Experience Scale (FIES) with eight items to measure household FI (19). The items evaluated in the ENPOVE 2022 were collected with a reference period of the previous month before the survey. The respondents were asked whether they or any adult member of their household had experienced one of the following situations (abbreviated item names in parentheses): (1) Did you worry that your household would run out of food? (WORRIED); (2) Were you unable to eat healthy and nutritious food or food that you preferred? (HEALTHY); (3) Did you eat only a few kinds of foods? (FEWFOOD); (4) Did you skip a meal because there was not enough food? (SKIPPED); (5) Did you eat less than you thought you should eat? (ATELLES); (6) Did your household run out of food? (RUNOUT); (7) Did you go hungry because you could not afford enough food? (HUNGRY); and (8) Did you go without eating for a whole day? (WHLDAY). All questions were dichotomous with Yes or No response options. For item 2, the assigned value of the response was inverted. However, this item was excluded from the FIES scale because it obtained a value greater than 1.5 in the infit statistic in the Rasch model (19). For our analysis a “Yes” response to a question was considered as 1, and “No” as 0. Subsequently, items 1, 3, 4, 5, 6, 7, and 8 were summed to obtain a variable that classified households with a score ≥ 4 as having moderate to severe FI, and those with a score of 0 to 3 as having no or low FI. It was not considered necessary to change the cut-off points or categories because the Food and Agriculture Organization (FAO) documents do not indicate this procedure of adjusting the cut-off points when removing an item from the scale and because other studies (17, 18), which also eliminated items due to high infit values, applied the cut-off points of the FAO protocol without modification.

#### 2.2.2. Explanatory variables

Based on a review of the literature and the availability of variables from the ENPOVE 2022 (10, 14, 16), the following independent variables were selected: gender (male, female), age group of the household head in years (15 to 29, 30 to 39, 40 to 49, 50 or more), higher education of the household head (yes, no), physical or psychological limitations of the household head (yes, no), whether the household head worked in the last week (yes, no), holding of a migratory permit by the household head (yes, no), time of arrival in Peru (6 or more years, less than or equal to 5 years), household members with health insurance (yes, no), rented housing (yes, no), wealth tercile (low, middle, high), presence of children under 5 years old in the household (no, yes), presence of elderly adults in the household (no, yes), household size (single-person, 2 to 5, 6 or more), and city of residence (Metropolitan Lima, Arequipa, Chiclayo, Chimbote, Ica, Piura, Trujillo, Tumbes).

We created the wealth tercile which took into consideration housing characteristics and household goods or services (20). Each characteristic (walls, roofs, floors, water, drainage and lighting, internet, television, stove, blender, iron, computer, cell phone, landline, radio, refrigerator and washing machine) was recategorized as a dichotomous variable (yes, no) assigning it a score generated through principal components analysis (PCA) and each household was assigned a score for each characteristic, and the scores for each household were summed (20). All results included the sample weights and households were classified according to the total score of the household and three equal categories (terciles) were created: “low,” “middle” and “high.”

### 2.3. Statistical analysis

Stata 17.0 (StataCorp, College Station, TX, United States) was used to clean, recode, and analyze the data. All analyses included the complex sampling characteristics and household sampling weights of the ENPOVE 2022 survey. The R programming language in the RStudio environment (R Core Team 2020) was used to evaluate the Rasch fit statistics (infit and outfit) and overall model fit for the FIES using the RM.weight package, and the results are presented for both the 8-item and 7-item (excluding item 2) versions of the FIES.

Summary statistics and cross-tabulations were used to describe the study sample. Chi-square tests with Rao-Scott correction were performed to determine differences between the proportions of the variables included in the study. The 95% confidence intervals (CIs) were estimated using the Taylor series linearization method. Poisson log generalized linear regression models were fitted to evaluate the association between independent variables and food insecurity, reporting prevalence ratios (PR) and 95% CIs as measures of association. The adjusted analysis included all independent variables with a value of p less than 0.20 obtained in the bivariate analysis. Multicollinearity of the independent variables was evaluated by the variance inflation factor (no multicollinearity was found). We also conducted sensitivity analyses to verify whether the results of the associated factors changed when the 8 FIES items were included. The sensitivity analyses are presented in the Supplementary Material. Finally, a value of p of less than 0.05 was considered statistically significant.

### 2.4. Ethical considerations

The ENPOVE 2022 databases do not contain information that would allow respondents to be identified. Since our study was based on the secondary data analysis of the ENPOVE 2022, which databases are freely and publicly available in the INEI microdata repository,^1^ ethical approval was not required.

## 3. Results

### 3.1. Characteristics of the study population

A total of 3,491 households in which Venezuelan migrants and refugees resided were included in the analysis. Regarding household heads, it was found that the majority were males [64.9% vs. females (35.1%)] and belonged to the age group of 30–39 years (38.9%). Further details of the sociodemographic, economic, and migratory characteristics of the population included are presented in Table 1.

**Table 1:** Characteristics of the households included in this study, ENPOVE 2022.

Characteristic	Absolute frequency (*n* = 3,491)	%^*^
Gender of household head		
Male	2,229	64.9
Female	1,262	35.1
Age group (years) of household head		
15–29	1,207	32.9
30–39	1,300	38.9
40–49	621	17.6
50 or more	363	10.6
Higher education of household head		
Yes	1,584	47.9
No	1907	52.1
Physical or psychological limitation of household head		
Yes	60	2.0
No	3,431	98.0
Household head worked in the last week		
Yes	3,001	86.6
No	490	13.4
Holding of a migratory permit by the household head		
Yes	2,364	75.1
No	1,127	24.9
Arrival in Peru of household head		
6 or more years	339	10.9
Less than or equal to 5 years	3,152	89.1
Household members with health insurance		
Yes	309	10.7
No	3,182	89.3
Rented house		
Yes	3,317	95.6
No	174	4.4
Wealth tercile		
Lowest	1,272	33.4
Middle	1,199	35
Highest	1,020	31.6
Presence of children under 5 years of age		
No	2,428	71.3
Yes	1,063	28.7
Presence of an older adult (60 and over)		
No	3,237	93.0
Yes	254	7.0
Household size		
Unipersonal	611	17.7
2–5	2,572	74.5
6 or more	308	7.8
City of household		
Lima Metropolitana	1922	83.8
Arequipa	204	3.6
Chiclayo	192	1.5
Chimbote	234	1.4
Ica	189	2.5
Piura	188	1.9
Trujillo	368	4.5
Tumbes	194	0.8

^*^The weighting factor and sample specifications of ENPOVE were included. ENPOVE, Encuesta Población Venezolana.

### 3.2. Proportion and rasch model of the FIES

**Figure 1:**
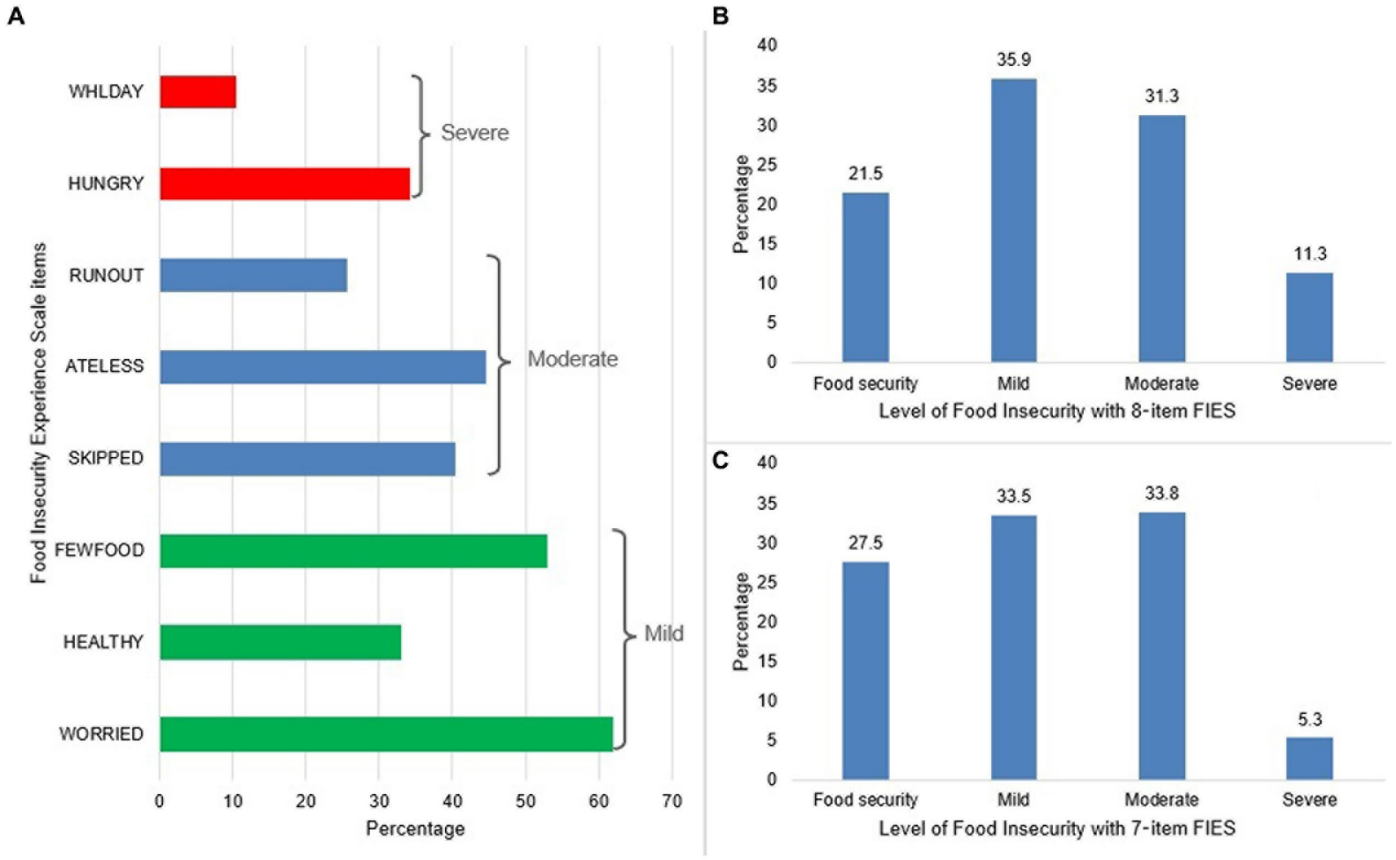
**(A)** Raw score of responses to Food Insecurity Experience Scale items and **(B)** and **(C)** level of food insecurity according to the 8- or 7-item Food Insecurity Experience Scale.

Figure 1A shows the proportion of affirmative responses to each of the 8 FIES items, where the lowest proportion was found in the WHLDAY category (10.5%), while the highest proportion was found in the WORRIED item (61.8%). Also, Figure 1B shows that the proportions of mild, moderate and severe food insecurity using the 8-item FIES were 35.9, 31.3, and 11.3%, respectively; while the 7-item FIES, which was used in this study, reported that mild, moderate and severe food insecurity were 33.5, 33.8, and 5.3%, respectively (Figure 1C).

All infit statistics for the 7 items included were within the widely acceptable range or in the range that can still be used for measurement (0.7–1.5), implying that they measured the same latent trait. Furthermore, the reliability of the Rasch model was 0.71, suggesting adequate consistency of the instrument (Table 2).

**Table 2:** Evaluation of the assumptions of the rash model of the Food Insecurity Experience Scale.

Item	Item severity	Standard error	Infit	Outfit
8 Items^*^				
WORRIED	−1.8689310	0.05363209	0.8903619	0.9094915
HEALTHY	0.3255702	0.05038797	1.7750747	2.7845104
FEWFOOD	−1.1897263	0.05076805	1.1033882	1.3289784
SKIPPED	−0.2541140	0.04969296	0.7857971	0.6801665
ATELESS	−0.5618956	0.04978105	0.7247948	0.6695117
RUNOUT	0.8991941	0.05258210	0.8873609	0.7962524
HUNGRY	0.2214051	0.05016552	0.6867110	0.5311893
WHLDAY	2.4381822	0.07043999	0.9700901	1.2260546
7 Items^**^				
WORRIED	−2.1755354	0.0628402	1.0398901	1.6554289
FEWFOOD	−1.3275513	0.05671906	1.3632707	2.1725721
SKIPPED	−0.2222556	0.05474767	0.8691253	0.8310948
ATELESS	−0.5834282	0.05491153	0.8115017	0.8314149
RUNOUT	1.1206663	0.05844651	0.9775228	0.9665989
HUNGRY	0.3338467	0.05535234	0.7125410	0.5675720
WHLDAY	2.8571335	0.08129745	1.0470332	2.2401959

^*^Rasch reliability: 0.73; Rasch reliability (flat): 0.72. ^**^Rasch reliability: 0.72; Rasch reliability (flat): 0.71.

### 3.3. Prevalence of moderate–severe food insecurity according to household characteristics

The prevalence of moderate–severe food insecurity (MSFI) was 39.0% (95% CI: 36.7–41.4%). The highest proportions of MSFI were observed in households in which the household head was female (46.7%), did not have a higher education (43.7%), had a physical or psychological limitation (53.4%), did not work in the last week (54.1%), did not have a migratory permit to reside in the city of residence (49.3%), and had arrived in Peru in the last 5 years (39.8%). Regarding household characteristics, the highest MSFI figures were found in households with at least one child under 5 years of age (42.8%), household members had health insurance (30.0%), it was a rented dwelling (39.5%), belonged to the lowest wealth index (52.7%), and were located in Tumbes (60.9%) and Chiclayo (52.7%) (Table 3).

**Table 3:** Frequency of moderate–severe food insecurity among Venezuelan households by background characteristics, ENPOVE 2022.

Characteristics	Moderate–severe food insecurity		
	No (*n* = 1,915) %^*^ (95% CI)	Yes (*n* = 1,576) %^*^ (95% CI)	*p*-value^**^
Overall	61.0 (58.6–63.3)	39.0 (36.7–41.4)	
Gender of household head			
Male	65.1 (62.2–67.9)	34.9 (32.1–37.8)	<0.001
Female	53.3 (49.5–57.1)	46.7 (42.9–50.5)	
Age group (years) of household head			
15–29	61.4 (57.5–65.2)	38.6 (34.8–42.5)	0.975
30–39	61.1 (57.5–64.6)	38.9 (35.4–42.5)	
40–49	60.7 (55.7–65.5)	39.3 (34.5–44.3)	
50 or more	59.7 (52.9–66.1)	40.3 (33.9–47.1)	
Higher education of household head			
Yes	66.1 (62.9–69.2)	33.9 (30.8–37.1)	<0.001
No	56.3 (53.0–59.4)	43.7 (40.6–47.0)	
Physical or psychological limitation of household head			
Yes	46.6 (31.0–62.8)	53.4 (37.2–69.0)	0.075
No	61.3 (58.8–63.6)	38.7 (36.4–41.2)	
Household head worked in the last week			
Yes	63.3 (60.7–65.8)	36.7 (34.2–39.3)	<0.001
No	45.9 (40.0–51.8)	54.1 (48.2–60.0)	
Holding of a migratory permit by the household head			
Yes	64.4 (61.6–67.0)	35.6 (33.0–38.4)	<0.001
No	50.7 (46.6–54.9)	49.3 (45.1–53.4)	
Arrival in Peru of household head			
6 or more years	67.4 (61.0–73.3)	32.6 (26.7–39.0)	0.035
Less than or equal to 5 years	60.2 (57.6–62.7)	39.8 (37.3–42.4)	
Household members with health insurance			
Yes	70.0 (62.9–76.3)	30.0 (23.7–37.1)	0.008
No	59.9 (57.4–62.4)	40.1 (37.6–42.6)	
Rented house			
Yes	60.5 (58.0–62.9)	39.5 (37.1–42.0)	0.029
No	71.9 (61.8–80.2)	28.1 (19.8–38.2)	
Wealth tercile			
Lowest	47.3 (43.4–51.2)	52.7 (48.8–56.6)	<0.001
Middle	61.7 (57.7–65.5)	38.3 (34.5–42.3)	
Highest	74.7 (71.0–78.0)	25.3 (22.0–29.0)	
Presence of children under 5 years of age			
No	62.5 (59.8–65.1)	37.5 (34.9–40.2)	0.024
Yes	57.2 (53.0–61.3)	42.8 (38.7–47.0)	
Presence of an older adult (60 and over)			
No	60.9 (58.4–63.4)	39.1 (36.6–41.6)	0.886
Yes	61.5 (53.9–68.6)	38.5 (31.4–46.1)	
Household size			
Unipersonal	64.5 (59.4–69.3)	35.5 (30.7–40.6)	0.115
2–5	60.7 (58.0–63.4)	39.3 (36.6–42.0)	
6 or more	55.1 (47.2–62.7)	44.9 (37.3–52.8)	
City of household			
Lima Metropolitana	61.5 (58.7–64.2)	38.5 (35.8–41.3)	<0.001
Arequipa	59.0 (50.6–67.0)	41.0 (33.0–49.4)	
Chiclayo	47.3 (39.2–55.6)	52.7 (44.4–60.8)	
Chimbote	56.5 (48.0–64.5)	43.5 (35.5–52.0)	
Ica	72.8 (64.6–79.7)	27.2 (20.3–35.4)	
Piura	56.3 (48.2–64.1)	43.7 (35.9–51.8)	
Trujillo	58.3 (51.9–64.4)	41.7 (35.6–48.1)	
Tumbes	39.1 (30.4–48.5)	60.9 (51.5–69.6)	

Data are displayed as weighted % of the row unless otherwise indicated. ^*^ The weighting factor and sample specifications of ENPOVE were included. ^**^ Estimated *p*-value using the Chi-square test with Rao-Scott adjustment. ENPOVE, Encuesta Población Venezolana; CI, Confidence Interval.

### 3.4. Determinants of moderate–severe food insecurity

In the adjusted analysis, it was observed that the female sex of the household head (adjusted PR [aPR]: 1.24; 95% CI: 1.11–1.39; *p* < 0.001), not having higher education (aPR: 1.12; 95% CI: 1.01–1.25; *p* = 0.041), not having worked in the last week (aPR: 1.27; 95% CI: 1.12–1.45; *p* = 0.001), household size consisting of 2 to 5 (aPR: 1.17; 95% CI: 1.01–1.37; *p* = 0.042), and 6 or more members (aPR: 1.38; 95% CI: 1.10–1.72; *p* = 0.005), and the household being located in Chiclayo (aPR: 1.24; 95% CI: 1.06–1.46; *p* = 0.008) and Tumbes (aPR: 1.21; 95% CI: 1.05–1.40; *p* = 0.008) increased the probability of experiencing MSFI in the household, while belonging to the middle (aPR: 0.74; 95% CI: 0.66–0.84; *p* < 0.001) and high wealth tertile (aPR: 0.50; 95% CI: 0.42–0.58; *p* < 0.001), and home located in Ica (aPR: 0.69; 95% CI: 0.53–0.90; *p* = 0.007) decreased the probability of this outcome (Table 4).

**Table 4:** Factors associated with moderate–severe food insecurity among Venezuelan households, ENPOVE 2022.

Variable	Crude		Adjusted^*^	
	PR (95% CI)	*p*-value	aPR (95% CI)	*p*-value
Gender of household head				
Male	Reference		Reference	
Female	1.34 (1.20–1.50)	<0.001	1.24 (1.11–1.39)	<0.001
Age group (years) of household head				
15–29	Reference		Not included	
30–39	1.01 (0.89–1.15)	0.902		
40–49	1.02 (0.87–1.19)	0.830		
50 or more	1.04 (0.86–1.26)	0.662		
Higher education of household head				
Yes	Reference		Reference	
No	1.29 (1.15–1.44)	<0.001	1.12 (1.01–1.25)	0.041
Physical or psychological limitation of household head				
Yes	Reference		Reference	
No	0.72 (0.53–0.99)	0.043	0.79 (0.57–1.10)	0.170
Household head worked in the last week				
Yes	Reference		Reference	
No	1.48 (1.30–1.68)	<0.001	1.27 (1.12–1.45)	<0.001
Holding of a migratory permit by the household head				
Yes	Reference		Reference	
No	1.38 (1.24–1.54)	<0.001	1.09 (0.97–1.22)	0.161
Arrival in Peru of household head				
6 or more years	Reference		Reference	
Less than or equal to 5 years	1.22 (1.01–1.49)	0.045	1.02 (0.85–1.24)	0.817
Household members with health insurance				
Yes	Reference		Reference	
No	1.34 (1.06–1.68)	0.013	1.05 (0.83–1.32)	0.700
Rented house				
Yes	Reference		Reference	
No	0.71 (0.51–0.99)	0.045	0.78 (0.57–1.08)	0.140
Wealth tercile				
Lowest	Reference		Reference	
Middle	0.73 (0.65–0.82)	<0.001	0.74 (0.66–0.84)	<0.001
Highest	0.48 (0.41–0.56)	<0.001	0.50 (0.42–0.58)	<0.001
Presence of children under 5 years of age				
No	Reference		Reference	
Yes	1.14 (1.02–1.28)	0.021	1.05 (0.94–1.19)	0.372
Presence of an older adult (60 and over)				
No	Reference		Not included	
Yes	0.99 (0.81–1.21)	0.886		
Household size				
Unipersonal	Reference		Reference	
2–5	1.11 (0.95–1.29)	0.186	1.17 (1.01–1.37)	0.042
6 or more	1.27 (1.01–1.58)	0.038	1.38 (1.10–1.72)	0.005
City of household				
Lima Metropolitana	Reference		Reference	
Arequipa	1.06 (0.86–1.32)	0.573	1.21 (0.98–1.49)	0.082
Chiclayo	1.37 (1.15–1.63)	<0.001	1.24 (1.06–1.46)	0.008
Chimbote	1.13 (0.92–1.39)	0.239	0.96 (0.78–1.17)	0.661
Ica	0.71 (0.53–0.94)	0.018	0.69 (0.53–0.90)	0.007
Piura	1.13 (0.93–1.38)	0.211	1.05 (0.88–1.25)	0.607
Trujillo	1.08 (0.92–1.28)	0.344	1.04 (0.89–1.22)	0.598
Tumbes	1.58 (1.34–1.87)	<0.001	1.21 (1.05–1.40)	0.008

Weighting factors and sample specifications of ENPOVE were included for all analysis. ENPOVE, Encuesta Población Venezolana; PR, Prevalence Ratio; aPR, Adjusted Prevalence Ratio; CI, Confidence Interval.

### 3.5. Sensitivity analysis

When including the 8 FIES items in the calculation of the FI, the results of the associated factors found in the main analysis and the sensitivity analysis were largely consistent (see Supplementary Material). We only found not having higher education which was significant in the main analysis became non-significant in the sensitivity analysis, and the city of Arequipa which was not significant in the main analysis became significant in the sensitivity analysis. On the other hand, the prevalence of moderate–severe FI with 7 items went from 39.0% (95% CI: 36.7–41.4%) to 42.6% (95% CI: 40.2–45.1%) with the 8 items.

## 4. Discussion

This study sought to determine the prevalence and determinants of MSFI in the households of Venezuelan migrants and refugees in Peru. In addition, the reliability of the FIES as a tool to measure FI in the target population was determined. The findings of the present study showed that four out of ten households in which Venezuelan migrants and refugees resided experienced MSFI. The determinants associated with this outcome were related to socio-demographic characteristics of the household head and economic and geographic characteristics of the household. Regarding the FIES, our study showed that this tool had adequate internal consistency, and that its items assessed the same latent range. Additionally, the proportion of positive responses on the FIES items ranged from 10.5% for the WHLDAY item to 61.8% for the WORRIED item.

We found that approximately 40% of households in which Venezuelan migrants and refugees reside experienced MSFI. This finding is lower than that reported in studies of Venezuelan migrants and refugees residing in Trinidad and Tobago (86.61%) (16), in migrants and refugees from the Middle East and North Africa residing in the United States (40–71%) (21), in migrants from Haiti residing in Chile (78%) (22), and in undocumented migrant households in the United Kingdom (94.6%) (23), while it was higher than that reported in studies conducted in Libyan migrant families in Australia (13.7%) (11) and in migrants residing in Portugal (24). The differences between the prevalence of FI reported in our study and studies conducted in various regions of the world could be due to the use of the instruments (Latin American and Caribbean Food Security Scale [ELCA], United States Department of Agriculture Household Food Security Survey Module [USDA HFSSM], and Ten-item Radimer/Cornell Hunger Scale) that differ from the FIES and the unit of analysis, since the studies conducted in Trinidad and Tobago, the United States, Chile and Portugal assessed FI at the individual level. Only the study conducted in Trinidad and Tobago used the FIES as a tool to measure FI (16). However, the temporality (during the COVID-19 pandemic) used in this study was different from ours, which would generate a higher proportion of FI. Although the differences found between the studies could generate dissimilar proportions of FI, these figures expose a global problem in vulnerable populations (such as migrants and refugees). Specifically, in Peru, our result is lower than that reported in Venezuelan migrant and refugee households during the COVID-19 pandemic (76.3%) (9). However, this difference could be influenced by the timing of the study (conducted during the COVID-19 pandemic) (9), during which there were higher unmet basic needs in health and food due to the social isolation during the pandemic and the prioritization of economic resources in health strategies. In addition, the report from Peru used the database of a non-governmental organization as a sampling frame and included beneficiaries of an intervention in districts of Metropolitan Lima with low socio-economic status, while the present study represented migrants in urban areas at the national level. Thus, our finding could expose situations of vulnerability that have developed as a consequence of the preventive measures put in place, the constant food shortages and prioritization of resources during the pandemic (25), which may have aggravated the nutritional and FI status of the households in which migrants reside.

Regarding the determinants associated with MSFI, it was found that socio-demographic determinants of the head of household such as being female, not having a higher education and not having worked in the last week increased the probability of MSFI. In addition to these determinants, economic and geographic characteristics of the household such as household size of 2 or more members and being located in Chiclayo and Tumbes were reported to increase the probability of this outcome, while a medium and high wealth index, and the household being located in Ica decreased the probability of this outcome. Our findings are consistent with those reported in studies of Venezuelan migrants and refugees in Trinidad and Tobago (16), Libyan migrant families in Australia (11) and migrant and refugee households in Colombia (26). With respect to the sex of the household head, female-headed households have greater economic challenges and disadvantages related to lower incomes, higher levels of informal work (housecleaning or street vending) and a greater dedication to household chores, which may impact FI (26). Household heads with lower levels of education have a higher prevalence of FI because education is associated with lower employment rates and, consequently, lower income, which hinders access to food with high nutritional value (27, 28). In addition, households with two or more members experience FI due to higher food costs that result in higher living expenses to ensure adequate nutrition in large households (29). Similarly, households located in Chiclayo and Tumbes experience higher levels of MSFI, which could be attributed to the large number of migrants located in these cities (30). Tumbes is a border region where the highest numbers of Venezuelan migrants enter, while Chiclayo is characterized by better job opportunities compared to other regions, which could be one of the reasons for the high migration of Venezuelans to this region (30). However, the migrant population in these cities lives in conditions of poverty, discrimination and unsatisfied basic needs.

On the other hand, the socio-economic level of households is a determining factor for experiencing FI. According to the biomedical literature, the economic income of migrants influences their food choices and dietary diversity, since lower purchasing power is associated with the consumption of high-calorie, low-cost foods, as well as lower dietary diversity (10, 31), which would have an impact on an inadequate diet and a higher prevalence of FI. It is worth mentioning that many Venezuelan migrants and refugees have experienced FI prior to their emigration due to lower income (8). Thus, our findings suggest that in Peru, these figures may have increased due to the unfavorable conditions experienced by migrants. Also, households located in Ica presented lower MSFI figures, which could be attributed to the fact that Ica has one of the fastest growing economies at the national level and would provide employment opportunities for migrants in the manufacturing and agricultural sectors, among others (32). These characteristics of the labor market have an impact on household incomes and increase access to food with a high nutritional content.

To determine the FI in our target population, we used the FIES, a widely used tool that was developed and validated internationally by the FAO (19, 33). According to our findings, this tool was found to have adequate infit statistics and internal consistency after excluding the HEALTHY item for having a value greater than 1.5. In addition, the proportions of positive responses to the FIES were: 61.8% (WORRIED), 52.9% (FEWFOOD), 40.5% (SKIPPED), 44.6% (ATELESS), 25.7% (RUNOUT), 34.3% (HUNGRY), and 10.5% (WHLDAY). The figures for items 1 to 5 are higher than those obtained for RUNOUT, HUNGRY and WHLDAY, which would indicate an adequate order of severity, and items 6 to 8 obtained the lowest proportions, as reported in previous studies recommending their use as global anchors (33). These results highlight their usefulness for estimating FI in Venezuelan migrant and refugee households.

Our findings have implications for the design and implementation of health policies. First, governmental and non-governmental institutions should consider the main determinants that increase the likelihood of FIMS as starting points for strategy or policy formulation. Second, non-governmental institutions (such as the United Nations Refugee Agency) that provide humanitarian support to Venezuelan migrants and refugees should prioritize resources to serve the population currently suffering from FI or at increased risk of experiencing IF and living in unfavorable conditions. Finally, government institutions should redouble efforts to improve the living and nutritional conditions of migrants through the granting of migration permits, improvements in working conditions, nutrition education campaigns, and improvements in access to and availability of foods with high nutritional value to achieve dietary diversity and adequate nutritional indicators. These changes could impact the fulfilment of SDG 2.1, which seeks to ensure access to food for all populations, including the most vulnerable (34).

The main strength of our study is the use of a database with complex sampling, representative at the Peruvian level and including many Venezuelan migrants and refugees residing in the main cities of Peru, which provides a current socio-demographic, economic and nutritional overview of one of the populations experiencing the greatest situations of vulnerability. In addition, the determination of FI was carried out using the FIES scale, which is a tool developed and validated internationally by the FAO, and which has been used for the study of FI worldwide during the pandemic period (9, 19). However, the present study is not without limitations. First, the lack of temporality in the measurement of the variables prevents causality from being established. Second, the interpretation of the FIES items may not have been correctly understood by respondents due to the negative or positive orientation of the items. Third, there is a possible recall bias, because some variables were based on events that occurred at specific points in the past, although the study period was shortened to the last 30 days to reduce this bias. Finally, some variables, such as the receipt of humanitarian support, economic bonuses or income by main or secondary occupation, which have not been included in this study due to their unavailability in the database.

In conclusion, four out of ten Venezuelan migrant and refugee households experienced MSFI. The determinants that increase the prevalence of MSFI are related to the socio-demographic characteristics of the head of household and economic and geographic characteristics of the household. In this sense, the institutions in charge of ensuring adequate quality and quantity of food should carry out public health strategies focused on the main determinants that influence the appearance of FI in Venezuelan migrants and refugees, especially when this population experiences poorer conditions of access to health, education, food and employment. Furthermore, this study is a starting point for epidemiological studies to assess dietary quality, nutritional indicators and dietary diversity in one of the most vulnerable populations in Peru.

## Data availability statement

Publicly available datasets were analyzed in this study. The datasets analyzed for this study can be found on the website of the Instituto Nacional de Estadística e Informática (http://iinei.inei.gob.pe/microdatos/).

## Author contributions

AH-V: conceptualization, data curation, formal analysis, investigation, methodology, software, supervision, validation, and writing – original draft. AH-V, JA, and RV-F: project administration. AH-V, RV-F, FV-L, and JA: visualization. AH-V, RV-F, and FV-L: writing – review and editing. All authors contributed to the article and approved the submitted version.

## Conflict of interest

The authors declare that the research was conducted in the absence of any commercial or financial relationships that could be construed as a potential conflict of interest.

## Publisher’s note

All claims expressed in this article are solely those of the authors and do not necessarily represent those of their affiliated organizations, or those of the publisher, the editors and the reviewers. Any product that may be evaluated in this article, or claim that may be made by its manufacturer, is not guaranteed or endorsed by the publisher.

## Footnotes

^1^https://proyectos.inei.gob.pe/microdatos/
