# Supplementary material for: Prevalence and socioeconomic determinants of food insecurity among Venezuelan migrant and refugee urban households in Peru
Source: Front Nutr. 2023 Jun 15;10:1187221. doi: 10.3389/fnut.2023.1187221 (PMC10308025; doi:10.3389/fnut.2023.1187221)
Supplement: Supplementary file 1 [file Table_1.docx]

Supplementary Material

**Prevalence and socioeconomic determinants of food insecurity among Venezuelan migrant and refugee urban households in Peru**

Akram Hernández-Vásquez*, Rodrigo Vargas-Fernández, Fabriccio J. Visconti-Lopez, Juan Pablo Aparco

*** Correspondence:** Akram Hernández-Vásquez: [ahernandez@usil.edu.pe](mailto:ahernandez@usil.edu.pe)

# Supplementary Tables

**Table S1.** Factors associated with moderate-severe food insecurity measured with 8-items FIES in Venezuelan households, ENPOVE 2022.

| **Variable** | **Crude** | |  | **Adjusted*** | |
| --- | --- | --- | --- | --- | --- |
|  | **PR (95% CI)** | **P-value** |  | **aPR (95% CI)** | **P-value** |
| Gender of household head |  |  |  |  |  |
| Male | Reference |  |  | Reference |  |
| Female | 1.31 (1.18-1.45) | <0.001 |  | 1.22 (1.10-1.35) | <0.001 |
| Age group (years) of household head |  |  |  |  |  |
| 15-29 | Reference |  |  | Not included |  |
| 30-39 | 1.02 (0.91-1.15) | 0.695 |  |  |  |
| 40-49 | 1.06 (0.92-1.22) | 0.429 |  |  |  |
| 50 or more | 0.98 (0.82-1.18) | 0.872 |  |  |  |
| Higher education of household head |  |  |  |  |  |
| Yes | Reference |  |  | Reference |  |
| No | 1.24 (1.12-1.37) | <0.001 |  | 1.08 (0.98-1.20) | 0.114 |
| Physical or psychological limitation of household head |  |  |  |  |  |
| Yes | Reference |  |  | Reference |  |
| No | 0.71 (0.54-0.94) | 0.015 |  | 0.77 (0.57-1.03) | 0.075 |
| The household head works |  |  |  |  |  |
| Yes | Reference |  |  | Reference |  |
| No | 1.42 (1.25-1.60) | <0.001 |  | 1.24 (1.09-1.40) | 0.001 |
| Holding of a migratory permit by the household head |  |  |  |  |  |
| Yes | Reference |  |  | Reference |  |
| No | 1.35 (1.22-1.50) | <0.001 |  | 1.09 (0.98-1.21) | 0.130 |
| Arrival in Peru of household head |  |  |  |  |  |
| 6 or more years | Reference |  |  | Reference |  |
| Less than or equal to 5 years | 1.26 (1.04-1.51) | 0.016 |  | 1.06 (0.89-1.27) | 0.506 |
| Household members with health insurance |  |  |  |  |  |
| Yes | Reference |  |  | Reference |  |
| No | 1.33 (1.08-1.64) | 0.008 |  | 1.05 (0.85-1.30) | 0.659 |
| Rented house |  |  |  |  |  |
| Yes | Reference |  |  | Reference |  |
| No | 0.71 (0.52-0.97) | 0.032 |  | 0.79 (0.58-1.07) | 0.129 |
| Wealth tercile |  |  |  |  |  |
| Lowest | Reference |  |  | Reference |  |
| Middle | 0.78 (0.70-0.87) | <0.001 |  | 0.79 (0.70-0.88) | <0.001 |
| Highest | 0.51 (0.44-0.59) | <0.001 |  | 0.52 (0.45-0.60) | <0.001 |
| Presence of children under 5 years of age |  |  |  |  |  |
| No | Reference |  |  | Reference |  |
| Yes | 1.12 (1.01-1.24) | 0.029 |  | 1.04 (0.93-1.15) | 0.519 |
| Presence of an older adult (60 and over) |  |  |  |  |  |
| No | Reference |  |  | Not included |  |
| Yes | 0.97 (0.81-1.17) | 0.769 |  |  |  |
| Household size |  |  |  |  |  |
| Unipersonal | Reference |  |  | Reference |  |
| 2-5 | 1.16 (1.01-1.34) | 0.038 |  | 1.23 (1.06-1.43) | 0.005 |
| 6 or more | 1.25 (1.01-1.55) | 0.042 |  | 1.36 (1.10-1.69) | 0.004 |
| City of household |  |  |  |  |  |
| Lima Metropolitana | Reference |  |  | Reference |  |
| Arequipa | 1.11 (0.91-1.34) | 0.294 |  | 1.24 (1.03-1.50) | 0.025 |
| Chiclayo | 1.38 (1.17-1.63) | <0.001 |  | 1.26 (1.08-1.46) | 0.003 |
| Chimbote | 1.19 (1.00-1.42) | 0.056 |  | 1.02 (0.85-1.22) | 0.820 |
| Ica | 0.77 (0.59-0.99) | 0.044 |  | 0.76 (0.59-0.96) | 0.023 |
| Piura | 1.14 (0.95-1.36) | 0.149 |  | 1.06 (0.90-1.25) | 0.469 |
| Trujillo | 1.09 (0.94-1.27) | 0.257 |  | 1.06 (0.92-1.22) | 0.454 |
| Tumbes | 1.49 (1.28-1.74) | <0.001 |  | 1.18 (1.03-1.35) | 0.016 |

Weighting factors and sample specifications of ENPOVE were included for all analysis. ENPOVE: Encuesta Población Venezolana. PR: Prevalence Ratio. aPR: Adjusted Prevalence Ratio. CI: Confidence Interval.
